# Model of Anti-Mullerian Hormone Over Age to Predict Menopause in Polycystic Ovary Syndrome and Eumenorrheic Women: A Study on Southern Indian Population

**DOI:** 10.7759/cureus.43419

**Published:** 2023-08-13

**Authors:** Hassan Mohammad, Janaki C S, Geetha Haripriya, Dinesh Maskeri, Prabhu K, Padma Priya

**Affiliations:** 1 Anatomy, Northern Border University, Arar, SAU; 2 Anatomy, Bharath Medical College and Hospital, Chennai, IND; 3 Gynecology, Prashanth Hospital, Chennai, IND; 4 Physiology, Anna Medical College, Montagne Blanche, MUS; 5 Anatomy, Sree Balaji Medical College and Hospital, Chennai, IND; 6 Microbiology, Bharath Medical College, Chennai, IND

**Keywords:** least squares model, menopause, ovarian reserve, polycystic ovarian syndrome, anti-mullerian hormone

## Abstract

Introduction

Anti-mullerian hormone (AMH) is a member of the transforming growth factor β family and is a marker of ovarian reserve; it is produced by the granulosa cells of developing preantral and early antral ovarian follicles. AMH concentration decreases with increasing age. Its concentration is increased relatively in those with polycystic ovary syndrome (PCOS) than eumenorrheic women.

Objectives

In this research, using a model of AMH over age, the age of menopause is predicted in PCOS and eumenorrheic women.

Study design

The study subjects were classified into two groups. Group 1 included PCOS subjects. Group 2 included eumenorrheic women. General profiles such as age, BMI, and hormonal parameters such as AMH were measured. Based on the exponential functional dependence of AMH over age, a model was proposed, and the value of exponential constants such as log βo and β1 were calculated using least square approximation. An arbitrary value of 0.2 ng/mL for AMH was taken as a cut-off value to predict the age of menopause in both groups.

Outcome measures

We predicted the age of menopause using a model of AMH over age by the least square approximation technique.

Results

The age prediction for menopause using the least squares model of AMH over age showed that in group 1 patients with PCOS, menopause is projected to occur at around 54.7 years, while in group 2 with eumenorrheic women, it is expected to happen at approximately 45.2 years.

Conclusion

Data demonstrates that serum AMH concentration declines over time, and predicting the age of menopause reflects sustained reproductive life span in PCOS subjects.

## Introduction

The human ovary is onset with early senescence and is characterized by the gradual decline in both the quantity and quality of oocytes residing within the follicles. The basic change of reproductive senescence in women is ovarian follicle loss. The total number of primordial follicles in the ovarian pool is called “ovarian reserve” (OR). The quantity of primordial follicles at various ages is reported by various studies [1-3]. At present, there is no accurate marker or clinical procedure to determine the quantity of primordial follicular pools in the ovary. Ultrasonographic measurement of ovarian volume and antral follicular count (AFC), and baseline hormonal parameters such as follicle-stimulating hormone (FSH) and estradiol indirectly, whereas hormones such as anti-mullerian hormone (AMH) and inhibin directly assess the OR of the ovary. AMH is synthesized by the granulosa cells of ovary small follicles and can inhibit small follicle maturation [4], demonstrating superiority in predicting OR and stimulation response [4-6].

In both human and animal studies [7], it has been proposed that serum AMH concentration reflects the size of the primordial follicle pool and is considered as a marker for OR. AMH is a member of the transforming growth factor β superfamily and is secreted by the preantral and tiny antral follicles [8-9]. In women, as age advances, there is a decrease in the number of primordial follicles, accompanied by a corresponding decline in serum concentration of AMH [10]. It is also recognized as a good indicator of fertility potential, thus reflecting the ovarian reproductive age. The level of AMH appears to fluctuate much less over the menstrual cycle [11] and is also cycle independent when compared to other hormones [12]. Its concentration has been shown to decline with aging [13]. This biomarker shows how normo-ovulatory women's reproductive ability gradually declines with age [14].

In polycystic ovary syndrome (PCOS) the level of AMH is higher [15-17]. AMH is secreted in greater quantities in PCOS patients due to a growing amount of tiny ovarian follicles [15]. According to certain studies, people with PCOS may have a longer reproductive life span [16-17]. This hypothesis was backed up by cohort data [18-19]. Based on these concepts study, a model was proposed using the least square fit of AMH over time to predict menopause in PCOS subjects and eumenorrheic women as a prediction of menopause, which has increasing clinical value as women postpone childbirth, and it helps to determine the reproductive age period.

## Materials and methods

The present work was approved by the Institutional Ethical Committee of Sree Balaji Medical College, and informed consent was obtained from all the patients. The subjects under study included women of childbearing age, who were divided into two groups. Group 1 included around 500 patients aged 30 to 35 years with body mass index (BMI) in the range of 24 to 31 with PCOS having amenorrhea (absence of vaginal bleeding for at least six months) or oligomenorrhea (interval between periods >35 days), with serum FSH concentrations within normal limits (1-10 IU/L). These patients were diagnosed as PCOS subjects based on hyperandrogenism and/or polycystic ovaries on ultrasound, per the Rotterdam ESHRE/ASRM-Sponsored PCOS Consensus Workshop Group 2004 [20].

Group 2 included 500 eumenorrheic subjects with inclusion criteria of regular menstrual cycle length (26-35 days), 30-35 years of age, BMI of 22-28 kg/m^2^, the absence of endocrine diseases or any other related disorder, and no use of medications or oral contraceptives during last three months prior to the induction to the study.

Under aseptic precaution, blood samples were obtained by venepuncture and processed within 2 hours after withdrawal; serum was separated and stored at 20°C until assayed. Serum AMH was assayed using the AMH/MIS enzyme-linked immunosorbent assay (ELISA) kit (Immunotech- Beckman, Marseille, France); the assay sensitivity was 0.7 pmol/L and the intra- and inter-assay coefficients of variation were 5.3% and 8.7%, respectively.

Statistical analysis

Association between age and AMH, as well as its changes over time, were measured. Based on the above functional dependence of AMH in the form of exponential relation with age, i.e., a model age = β0 (AMH) -β1 was proposed. The method shows the calculation of beta 0 and beta 1, and the value of coefficients are calculated using least squares approximation; this model will explain how least squares approximation can be used to predict menopause, which is the end stage of ovarian aging. The value of 0.2 ng/mL for AMH was used as the cut-off value to predict menopause in eumenorrheic and PCOS women. The relative de-acceleration rate over the years and also the age at which the group 1 and group 2 subjects reach menopause were found using the above least squares fit model.

## Results

**Figure 1:**
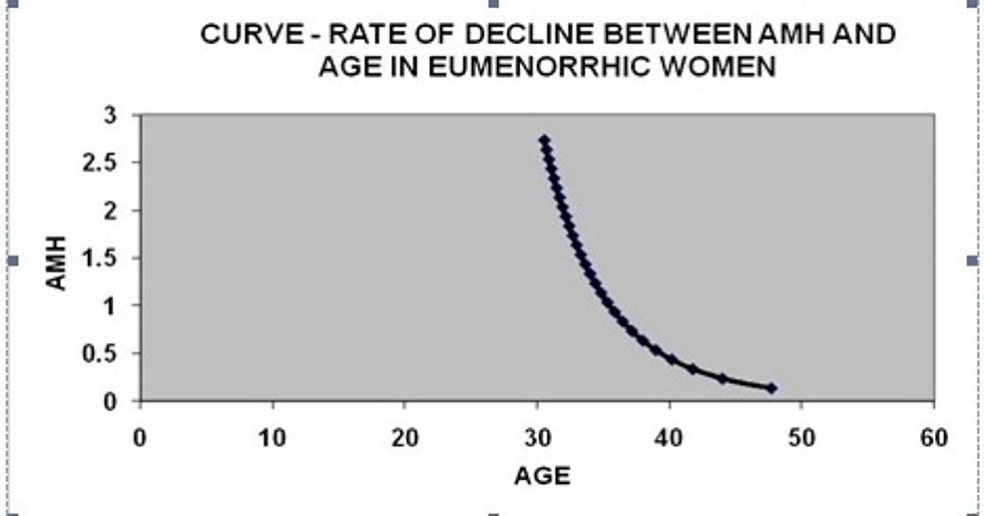
Curve rate of decline between AMH and age in eumenorrheic women. AMH and age are shown in the y-axis and x-axis, respectively. AMH, anti-mullerian hormone; PCOS, polycystic ovary syndrome

**Figure 2:**
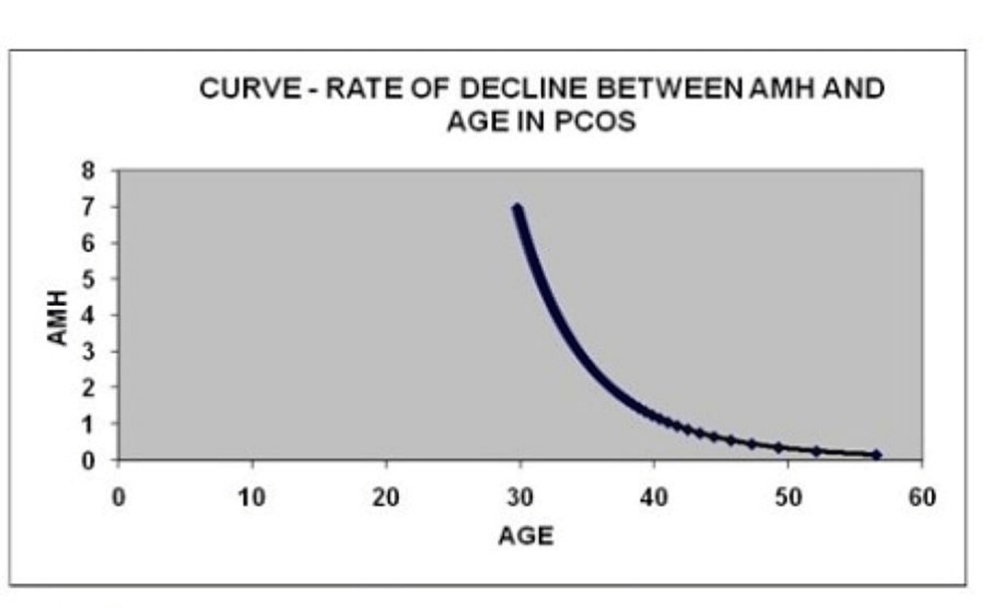
Curve rate of decline between AMH and age in PCOS women. AMH and age are shown in the y-axis and x-axis, respectively. AMH, anti-mullerian hormone; PCOS, polycystic ovary syndrome

In group 1, consisting of 500 patients aged between 30 and 35, with a BMI range of 24-31, the average age and average BMI were found to be 33.4 years and 27.5, respectively. This group primarily comprised women with PCOS. Group 2 included 500 eumenorrheic subjects aged 30-35 years, with BMI in the range of 22-28; the mean age and mean BMI were 34.3 years and 25.0, respectively. The mean value of AMH was 4.95 ng/mL in group 1 and 1.64 ng/mL in group 2 and was three times elevated in PCOS subjects. Table 1 presents the logarithmic values of the exponential constants indicating the relationship between AMH and age for the two groups. In group 1, log beta 0 was 41.41 and beta 1 was 0.17, while in group 2, log beta 0 was 35.49 and beta 1 was 0.15. The estimated mean age at which eumenorrheic subjects reach menopause was 45.2 years, while for PCOS patients, it was observed to be 54.7 years. Group 1 exhibited a prolonged reproductive life span. The curve rate between AMH and age in eumenorrheic women declined significantly, as shown in Figure 1; the curve rate between AMH and age in PCOS women is displayed in Figure 2. There is a decline in the AMH serum levels in both groups, but the curve in PCOS patients differed significantly from eumenorrheic women. An increased rate of de-acceleration in eumenorrheic women was observed in group 2 when compared with group 1 subject with PCOS condition. The information from the data suggests that both groups undergo a yearly decline in AMH concentrations. The curve in both groups shows that follicular atresia was biphasic and its de-acceleration was more in eumenorrheic women serving as group 2 than women with PCOS in group 1.

**Table 1:** The predicted model to determine the age of menopause in PCOS (group 1) and eumenorrheic (group 2) women. PCOS, polycystic ovary syndrome

Group		Log β_0_ value	Β_1_ value	Predicted age of menopause
Group 1	Predicted model age = β_0_(AMH)^- β1^	41.41	0.17	54.7
Group 2		35.49	0.15	45.2

## Discussion

This research indicates that the level of AMH was higher in PCOS subjects than eumenorrheic patients and that PCOS subjects seem to have extended reproductive life span as they reach menopause in late age when compared to the eumenorrheic subjects.

The basics of ovarian aging in women is the depletion of the primordial follicle pool [3], and the hallmark of follicle pool exhaustion is menopause. Critical elements in the process of ovarian aging include the number of primordial follicles present in the initial stock and the variables that control the rate at which this stockpile is lost. According to reports, 1,000 ovarian follicles are left in menopausal women, and the remaining primordial follicle pool is exhausted as women age reproductively [3]. The required quantity of primordial follicles, not age, decides when menopause will occur [21]. It was asserted that the rate of follicle loss increased from the age of 37.5 years till menopause [22] and that an abrupt change in follicular dynamics occurs when roughly 25,000 follicles are still present. According to these findings, the follicular pool is declining in a biphasic manner [22], with the rate of follicular degeneration beginning to shift or accelerate around the age of 37.5 years. The follicular distribution in the ovarian cortex is exceedingly diverse, as evidenced by other studies, which suggest that an ovarian biopsy sample is not typical of the true follicular pool, is unlikely to predict the OR, and is also an invasive test [23]. In order to emphasize serum AMH's reliability as a biological indicator of ovarian aging and its evidence for reproductive aging, attention has been drawn to it more recently.

AMH, unlike other indicators, is independent of the menstrual cycle and is a possible biomarker and predictor of poor OR, menopause, and early ovarian failure [24]. It does not change from cycle to cycle and has a strong intra-class correlation [25]. AMH plays a role in follicle development and has a strong relationship with chronological age, antral follicle count, and successful in vitro fertilization (IVF) [12]. This study showed that the drop in AMH with age was less pronounced in PCOS when compared to eumenorrheic and that serum AMH levels were higher in PCOS than in eumenorrheic subjects. The increased level of AMH in PCOS could be due to more median density of tiny follicles (including primordial and primary stage), which can be sixfold greater in anovulatory PCOS than in normal ovaries, or because these women may actually be born with a large OR [26], or due to over recruitment of follicles into the growing stages or slower progression during the later stage of follicle development [26-28].

The majority of women whose AMH level exceeded the arbitrary threshold of 0.2 ng/mL showed menopausal transition symptoms, such as unpredictable and irregular menstrual periods, demonstrating the validity of the cut-off level used to determine the age at which menopause had occurred. According to research on AMH levels in Iranian women in general, the average age of menopause was 49 years [19], and it was 49.6 years [29] for women who were normo-ovulatory. Conversely, the average PCOS patient experiences menopause two years later than women with normal ovulation, which may have clinical significance but is statistically insignificant. On the contrary, it was reported that the average menopausal ages were 74 and 42 years in normogonadotropic anovulatory and normo-ovulatory controls, respectively [18].

The predicted age of menopause in our model was found to be 54.5 years in PCOS and 45.2 in eumenorrheic subjects. For women everywhere, menopause begins between the ages of 45 and 55 years. The typical age of menopause in developed nations is around 51 years; however, it is believed to be 45.5 years in the Philippines, New Guinea, other parts of Africa, India, Pakistan, and Thailand [30]. However, our model showed statistical significance in our populations. Our model is sufficiently accurate to demonstrate that the actual ages at menopause in normo-ovulatory and PCOS patients were quite close to the predictions for those who reached menopause. This could be accomplished by giving PCOS patients more OR at birth in order to regulate the ovarian biological clock and maintain fertility in the face of unfavorable environmental conditions. As a result, it is important to keep in mind that each mathematical model has a set of biological implications that must be taken into account. It should also be noted that the prediction of a single model can differ significantly when plotted on other scales or altered axes. The age-specific AMH reference values reported in this study came from the southern Indian population. It is highlighted that other factors such as race and ethnicity [31], BMI [32], and PCOS [33] may have an impact on AMH. This is, as far as we are aware, the first report of its kind for this population.

According to the results of the current study, women who reach menopause by the age of 45 have seen their fertility fall more quickly. The current estimation of time of menopause could also identify those at higher risk of cardiovascular diseases, osteoporosis, and breast and endometrial cancer due to early or late menopause. The prediction of menopause is also for counseling the subjects in infertility treatment to choose the mode of treatment such as oocyte preservation and IVF. As reported previously, the rate of deceleration was biphasic, i.e.., from puberty to a critical point in which the follicle number decreased from 1 million to 25,000 and from critical point to menopause that is from 25,000 to 1,000. The above study also highlights the rate of deceleration is unsteady and steep from puberty [22] to the critical point and steady and slow from the critical point to menopause.

## Conclusions

In conclusion, this study conducted on the southern Indian population highlights the extended reproductive life span in PCOS women, who are predicted to reach menopause later than eumenorrheic women; in addition, the rate of decay was unsteady in both groups.
